# Antimicrobial agents and microbial ecology

**DOI:** 10.3934/microbiol.2022001

**Published:** 2022-01-06

**Authors:** Patrick Di Martino

**Affiliations:** Groupe Biofilm et Comportement Microbien aux Interfaces, Laboratoire ERRMECe Cergy Paris Université, 1 rue Descartes 95000 Neuville-sur-Oise, cedex, France

**Keywords:** Antimicrobial, antibiotic, disinfectant, resistance, ecology, biosourced, bioinspired

## Abstract

Antimicrobials are therapeutic substances used to prevent or treat infections. Disinfectants are antimicrobial agents applied to non-living surfaces. Every year, several thousand tonnes of antimicrobials and their by-products are released into the environment and in particular into the aquatic environment. This type of xenobiotic has ecological consequences in the natural environment but also in technological environments such as wastewater treatment plants and methane fermentation sewage sludge treatment plants. The constant exposure of microbial communities not only to high concentrations but also to sub-inhibitory concentrations of antibiotics is a key element in the development of antibiotic resistance in aquatic environments and in soils. The future of antimicrobials lies in the development of biosourced or bioinspired molecules. The observation and deciphering of interactions between living organisms is the key to this development.

Antimicrobials are therapeutic substances used to prevent or treat infections. They include antiseptics, antibiotics, antivirals, antifungals and antiparasitics. Disinfectants are antimicrobial agents applied to non-living surfaces. Antimicrobials can kill microorganisms and/or prevent their growth by targeting key steps in cellular metabolism such as the synthesis of biological macromolecules, the activity of cellular enzymes, or cellular structures such as the cell wall, cell membranes [1]–[4]. The presence of antimicrobial agents in an ecosystem, whether natural or man-made, always has an ecological impact [5].

Every year, several thousand tonnes of antimicrobials and their by-products are released into the environment and in particular into the aquatic environment [6]. In addition, some antimicrobials are particularly persistent in the environment, which facilitates their diffusion and accumulation in different compartments. This type of xenobiotic has ecological consequences in the natural environment but also in technological environments such as wastewater treatment plants and methane fermentation sewage sludge treatment plants [7]–[9]. The constant exposure of microbial communities not only to high concentrations but also to sub-inhibitory concentrations of antibiotics is a key element in the development of antibiotic resistance in aquatic environments and in soils [5],[10]. This leads to the development of antibiotic-resistant bacteria and to the spread of genetic determinants of resistance. In this context, environmental biofilms and their associated virome serve as reservoirs for antimicrobial resistance [10]. The development of nanoparticles with antibacterial activity is one of the solutions for combating bacteria that are multi-resistant to antibiotics [11].

Chlorine-based treatments are widely used during drinking water production and distribution. The post-chlorine treatment survival and regrowth of microorganisms, due to microbial chlorine resistance and to microbial chlorine tolerance, can conduct to an increase of human exposure to waterborne pathogens [12]. Resistance has a genetic origin and is transmissible, whereas tolerance is linked to transient phenotypic adaptations, i.e., extracellular polymeric substances production and biofilm formation [13],[14]. Microbial resistance to chlorine also poses problems for wastewater treatment [15].

Antimicrobials have been present in nature for much longer than their use by humans. The study of interactions between different microorganisms, whether prokaryotes or eukaryotes, is an important source of new antimicrobial discovery [16]–[20]. Ecological knowledge of antimicrobial production conditions, functions and roles in an ecosystem is necessary for the discovery of new antimicrobials and their safe and effective use. This approach can also be used for man-made environments like fermented artisanal food products [21].

The microbial ecology of the human mouth and gastrointestinal tract is very complex. The use of broad-spectrum antimicrobial agents can lead to (i) proliferation of previously minor components of the microbiota, (ii) colonisation by saprophytic organisms, (iii) colonisation by antimicrobial resistant pathogens, which may increase the risk of disease. [22]–[24]. Cranberry can help prevent urinary tract infections and avoid the use of antibiotics [25],[26]. In contrast to antibiotic treatment, dietary cranberry supplementation does not appear to affect the faecal concentrations of thermotolerant coliforms, *Enterococci* spp. and *Lactobacilli* spp. in Wistar rats [27]. Thus, dietary use of cranberry would not disturb the microbial ecology of the intestinal tract.

Antimicrobials are micropollutants that have a strong impact on different ecosystems. Studies are needed to understand their ecological impact and learn how to control them. The future of antimicrobials lies in the development of biosourced or bioinspired molecules. The observation and deciphering of interactions between living organisms is the key to this development.
